# Chronic disease concordance within Indian households: A cross-sectional study

**DOI:** 10.1371/journal.pmed.1002395

**Published:** 2017-09-29

**Authors:** Shivani A. Patel, Preet K. Dhillon, Dimple Kondal, Panniyammakal Jeemon, Kashvi Kahol, Sathya Prakash Manimunda, Anil J. Purty, Ajit Deshpande, P. C. Negi, Sulaiman Ladhani, Gurudayal Singh Toteja, Vikram Patel, Dorairaj Prabhakaran

**Affiliations:** 1 Centre for Chronic Disease Control, Gurugram, Haryana, India; 2 Hubert Department of Global Health, Emory University, Atlanta, Georgia, United States of America; 3 Public Health Foundation of India, Gurugram, Haryana, India; 4 National Centre for Disease Informatics and Research, Indian Council of Medical Research, Bengaluru, India; 5 Pondicherry Institute of Medical Science, Kalapet, Puducherry; 6 Sri Aurobindo Institute of Medical Sciences, Indore, Madhya Pradesh, India; 7 Indira Gandhi Medical College, Shimla, Himachal, Pradesh, India; 8 Aga Khan Health Services, Mumbai, Maharashtra, India; 9 Indian Council of Medical Research, Aansari Nagar, New Delhi, India; 10 Department of Global Health and Social Medicine, Harvard Medical School, Boston, Massachusetts, United States of America; 11 Sree Chitra Tirunal Institute for Medical Sciences and Technology, Trivandrum, India; Stanford University, UNITED STATES

## Abstract

**Background:**

The household is a potentially important but understudied unit of analysis and intervention in chronic disease research. We sought to estimate the association between living with someone with a chronic condition and one’s own chronic condition status.

**Methods and findings:**

We conducted a cross-sectional analysis of population-based household- and individual-level data collected in 4 socioculturally and geographically diverse settings across rural and urban India in 2013 and 2014. Of 10,703 adults ages 18 years and older with coresiding household members surveyed, data from 7,522 adults (mean age 39 years) in 2,574 households with complete covariate information were analyzed. The main outcome measures were diabetes (fasting plasma glucose ≥ 126 mg/dL or taking medication), common mental disorder (General Health Questionnaire score ≥ 12), hypertension (blood pressure ≥ 140/90 mmHg or taking medication), obesity (body mass index ≥ 30 kg/m^2^), and high cholesterol (total blood cholesterol ≥ 240 mg/dL or taking medication). Logistic regression with generalized estimating equations was used to model associations with adjustment for a participant’s age, sex, education, marital status, religion, and study site. Inverse probability weighting was applied to account for missing data. We found that 44% of adults had 1 or more of the chronic conditions examined. Irrespective of familial relationship, adults who resided with another adult with any chronic condition had 29% higher adjusted relative odds of having 1 or more chronic conditions themselves (adjusted odds ratio [aOR] = 1.29; 95% confidence interval [95% CI] 1.10–1.50). We also observed positive statistically significant associations of diabetes, common mental disorder, and hypertension with any chronic condition (aORs ranging from 1.19 to 1.61) in the analysis of all coresiding household members. Associations, however, were stronger for concordance of certain chronic conditions among coresiding household members. Specifically, we observed positive statistically significant associations between living with another adult with diabetes (aOR = 1.60; 95% CI 1.23–2.07), common mental disorder (aOR = 2.69; 95% CI 2.12–3.42), or obesity (aOR = 1.82; 95% CI 1.33–2.50) and having the same condition. Among separate analyses of dyads of parents and their adult children and dyads of spouses, the concordance between the chronic disease status was striking. The associations between common mental disorder, hypertension, obesity, and high cholesterol in parents and those same conditions in their adult children were aOR = 2.20 (95% CI 1.28–3.77), 1.58 (95% CI 1.15–2.16), 4.99 (95% CI 2.71–9.20), and 2.57 (95% CI 1.15–5.73), respectively. The associations between diabetes and common mental disorder in husbands and those same conditions in their wives were aORs = 2.28 (95% CI 1.52–3.42) and 3.01 (95% CI 2.01–4.52), respectively. Relative odds were raised even across different chronic condition phenotypes; specifically, we observed positive statistically significant associations between hypertension and obesity in the total sample of all coresiding adults (aOR = 1.24; 95% CI 1.02–1.52), high cholesterol and diabetes in the adult-parent sample (aOR = 2.02; 95% CI 1.08–3.78), and hypertension and diabetes in the spousal sample (aOR = 1.51; 95% CI 1.05–2.17). Of all associations examined, only the relationship between hypertension and diabetes in the adult-parent dyads was statistically significantly negative (aOR = 0.62; 95% CI 0.40–0.94). Relatively small samples in the dyadic analysis and site-specific analysis call for caution in interpreting qualitative differences between associations among different dyad types and geographical locations. Because of the cross-sectional nature of the analysis, the findings do not provide information on the etiology of incident chronic conditions among household members.

**Conclusions:**

We observed strong concordance of chronic conditions within coresiding adults across diverse settings in India. These data provide early evidence that a household-based approach to chronic disease research may advance public health strategies to prevent and control chronic conditions.

**Trial registration:**

Clinical Trials Registry India CTRI/2013/10/004049; http://ctri.nic.in/Clinicaltrials/login.php

## Abstract 2

In a cross-sectional analysis, Shivani Patel and colleagues estimate the extent of chronic disease concordance within Indian households.

## Author summary

**Why was this study done?:**

- Prior research, largely set in high-income country settings, demonstrates the concordance of physical health and mental health outcomes among spouses and between parents and their adult children.
- These prior studies have examined neither disease concordance among coresiding adults who are not parent-child pairs or spouses nor the correspondence of different chronic conditions (e.g., husband’s diabetes status and wife’s common mental disorder status). Few have examined physical and mental health outcomes simultaneously.
- Understanding associations of shared and differing chronic conditions among all coresiding adults in households may shed light on new approaches to identify and treat chronic illness in low- and middle-income countries such as India.

**What did the researchers do and find?:**

- We examined 5 chronic disease conditions—hypertension, diabetes, obesity, common mental disorder, and high total blood cholesterol—in 7,522 adults living in 2,574 households in 4 diverse settings in India.
- We demonstrate that there is substantial concordance in diabetes, common mental disorder, and obesity among Indian adults residing in the same household regardless of relationship type.
- Correspondence across different chronic conditions was weaker.

**What do these findings mean?:**

- Adults who live with someone with diabetes, common mental disorder, or obesity are more likely to have that same condition.
- Addressing the burgeoning chronic disease burden in India will benefit from understanding and intervening upon mechanisms responsible for disease concordance within households in this setting.

## Introduction

Chronic conditions are now the biggest contributor to disability-adjusted life years across the globe [1], and morbidity due to these conditions has increased at a faster rate in South Asia as compared to the rest of the world over the past 20 years [2]. Alongside these major epidemiologic changes, the extended family system—in which relatives beyond the nuclear family reside with one another—remains salient in India: 50% of children reside in households with adults in addition to their parents [3], and 77% of the elderly reside with their married adult children [4]. Indians, therefore, from cradle to grave are likely to share a household environment and health-promoting resources with family members and also be exposed to one another’s lifestyle practices (e.g., tobacco use, diet). Genetically related household members—such as parents and children—may additionally share a similar hereditary predisposition to disease. Yet, most epidemiologic studies and public health interventions in India targeting chronic conditions currently focus on individuals or occasionally the community [5] and largely ignore the family unit despite its potential importance in understanding risk and designing sustainable interventions.

Indeed, the literature suggests the promise of reorienting the focus of chronic disease research from the individual to the family. Prior systematic reviews drawing largely on populations residing in high-income countries (HICs) demonstrate the concordance of cardiovascular risk factors [6,7] and mental health and health behaviors [7] among spousal dyads. Concordance of cardiometabolic conditions among parent-child (e.g., [8,9]) and sibling dyads (e.g., [9]) has also been observed in studies conducted in HICs. A small but growing body of literature in Asian countries has examined and found concordance of cardiometabolic conditions among spousal [10–12], parent-child [11–13], and sibling [12,14] dyads and concordance of health behaviors among spousal dyads [15].

Genetic, environmental, and interpersonal mechanisms are 3 types of highly plausible drivers of familial concordance of disease implicitly or explicitly considered by prior studies. Understanding the extent of genetic predisposition to certain disease conditions has been the goal of many family-based studies [8,11], although genetic explanations of disease concordance largely apply to parent-child and sibling dyads. Environmental factors can include shared household socioeconomic resources important for health [7,16], a common household diet, and the extrahousehold shared community milieu (e.g., built environment and cultural norms around physical activity). Finally, interpersonal influences include modeling of lifestyle factors such as physical activity, diet, and smoking [7,17,18]; “affective contagion” in which the moods of those around us influence our own [7]; and the stress of living with and caring for someone with a chronic condition [19–22]. In addition to these 3 mechanisms, assortative mating (largely applicable to spousal pairs) or other self-selection processes into households/families may impact the concordance of chronic disease within families.

We seek to build upon the existing literature to address 2 unresolved but important issues regarding chronic disease concordance in households. First, we are aware of no studies that have gone beyond the spousal, parent-child, and sibling pairs that comprise the nuclear family to investigate associations of chronic disease status among all household members. Most of the hypothesized pathways linking chronic conditions within families would also apply to individuals beyond the nuclear family who reside in the same household. Second, we are aware of no studies that have investigated the correspondence of different chronic conditions among household members (e.g., husband’s diabetes status and wife’s common mental disorder status). Because many chronic conditions have common behavioral and psychosocial risk factors, shared environmental or interpersonal factors that lead to the development of a specific chronic condition in 1 household member may lead to the development of another chronic condition in a coresiding household member. For example, diabetes, hypertension, obesity, and high cholesterol are cardiometabolic conditions that are impacted by physical activity and dietary intake [23], and much evidence links diabetes and depression [24]. Ignoring the correspondence between chronic conditions may underestimate the degree of household aggregation of disease. Understanding associations of shared and differing chronic conditions among all coresiding adults in households may shed light on new approaches to identify and treat chronic illness in India and other low- and middle-income countries (LMICs) where extended family households are prominent [25].

The extent to which the household as a unit may be effectively leveraged for mechanistic studies of prevention and interventions targeting chronic conditions in India will depend on whether there is indeed concordance of the same chronic conditions or correspondence of differing chronic conditions within household members. The overarching goal of this study was to test the hypothesis that living with any household member who has a chronic condition—diabetes, common mental disorder, hypertension, obesity, and/or high cholesterol—raises the risk of developing the same or another chronic condition. To explore this hypothesis, we conducted an analysis to examine whether living with someone with a chronic condition relates to one’s own chronic condition status in coresiding adults in households across 4 geographically and socioculturally diverse districts in India. In addition, we examined these associations among dyads of parents and their adult children and of spouses living in the same household.

## Methods

### Data source

We conducted a cross-sectional observational analysis of the baseline survey and laboratory data from the Diet and Lifestyle Interventions for Hypertension Risk Reduction through Anganwadi Workers and Accredited Social Health Activists study (DISHA study) [26]. DISHA is a community-based cluster randomized trial designed to test the effectiveness of a community health worker-led lifestyle behavior change on hypertension reduction. The baseline study was conducted in 2013 and 2014 to measure risk factors in 4 regionally and socioeconomically diverse districts located in Madhya Pradesh, Gujarat, Tamil Nadu, and Himachal Pradesh that were selected for the initial phase of the intervention. Participants were selected using a multistage cluster sampling design stratified by district. Dhar District, Madhya Pradesh (central India), is home to a predominantly indigenous (*Adivasi*) population and has poor road connectivity. Junagadh District, Gujarat (western India), is a rural plains setting, while the Mashobra District, Himachal Pradesh (northern India), is a rural hilly setting consisting of sparsely populated villages. Finally, the Puducherry, a union territory bordering Tamil Nadu (southern India), is an urban and coastal setting with relatively better public health infrastructure. The primary sampling units were randomly selected villages within the districts (9–12 villages per site; a total of 45 villages in the study), from which 120–150 households were randomly selected. At the household level, all adults over the age of 18 years were invited to participate in the survey. We exploited this feature of the sampling design to identify adults residing in the same household.

Of the 11,751 participants linkable to the household demographic roster, 10,703 participants had at least 1 coresiding household member enrolled in the study. Of participants with coresiding household members, 3,181 were excluded because of missing data on 1 or more outcomes. Thus, a total of 7,522 participants residing in 2,574 households with complete covariates and at least 2 sampled adults per household were analyzed in the primary analysis. We additionally examined associations of interest among adults with coresiding parents (1,660 dyads in 1,199 households) and spouses (1,598 dyads in 1,598 households). Dyads were identified through each participant’s relationship with the household head. The analysis of dyads was restricted to pairs of participants who were unambiguously identifiable as parent-adult child pairs or spouses through the participants’ relationship to the household head.

The study obtained ethics approval from the Centre for Chronic Disease Control’s ethics committee (#IRB00006330), as well as ethics committees of participating sites. Participants provided written informed consent prior to being surveyed and assessed. This study is reported as per STROBE guidelines (S1 Text).

### Chronic conditions

We analyzed 5 prevalent chronic conditions: diabetes (prior diagnosis by a physician, fasting plasma glucose ≥ 126 mg/dL, or taking medication [27]), common mental disorder (i.e., depressive and anxiety disorders, measured here using the General Health Questionnaire score ≥ 12 [28,29]), hypertension (prior diagnosis by a physician, blood pressure ≥ 140/90 mmHg, or taking medication [30]), obesity (body mass index ≥ 30 kg/m^2^ [31]), and high cholesterol (prior diagnosis by a physician, total blood cholesterol ≥ 240 mg/dL, or taking medication [32]). We also created a composite binary variable indicating the presence of at least 1 of the 5 chronic conditions.

Data collection took place at the participant’s home. Height was measured using a stadiometer with accuracy of 2 mm (Seca), weight was measured using a digital weighing scale with accuracy of 100 gm (Seca), and systolic and diastolic blood pressure was measured using an electronic blood pressure monitor (OMRON 7080). A 5-ml fasting blood sample was collected from participants reporting at least 8 hours of fasting. The sample was centrifuged in the field, and the resulting serum and plasma samples were then transported to a central laboratory in New Delhi at the Indian Council of Medical Research for biochemical analysis and storage. Fasting plasma glucose was assessed using the Enzymatic Colorimetric Assay method. The General Health Questionnaire, previously validated for detecting common mental disorders in the Indian setting [28,29], was translated into the local language.

### Sociodemographic covariates

Sociodemographic and behavioral data were collected through standard survey tools. For more details, please see the DISHA methods paper [26]. Individual age (continuously specified in years), sex (binary), education (years of schooling and college), marital status (married or unmarried), and family religion (Hindu or other) were included in the analysis as correlates of chronic conditions.

### Statistical analysis

The development of the statistical analysis plan is described in S2 Text. We first constructed a set of indicator variables for each participant describing whether any other individual (excluding self) in the household had a given chronic condition. We next estimated 3 sets of logistic regression models that included differing groups of participants defined by the type of relationship among household members. Each set of logistic regression models estimated the relative odds of having any chronic condition for individuals living with a household member with any chronic condition relative to individuals who were not living with a household member with a chronic condition (i.e., the odds ratio of any chronic condition associated with living with someone who has any chronic condition). In addition, we estimated the relative odds of a given chronic condition associated with living with a household member with that same chronic condition (chronic condition concordance; e.g., the odds ratio of diabetes that is associated with living with someone who has diabetes) and living with a household member with a different chronic condition (chronic condition correspondence; e.g., the odds ratio of diabetes that is associated with living with someone who has a common mental disorder).

The first set of models included data from all available household members aged 18 years and older and was agnostic to the type of relationship between the index participant and the coresiding household members. To examine whether associations were observed across all study sites, we also estimated a set of models with an interaction term between the exposure condition and the study site. We tested the statistical significance of the interaction term using generalized score tests for Type III contrasts.

A second set of models examined associations among adult children with coresiding parents. For this analysis, the exposure was the presence of a given chronic condition in either parent for whom we had data. A third set of models examined associations among spousal dyads. In the spousal analysis, the wife’s chronic condition status was modeled as the outcome, and the husband’s chronic condition status was considered the exposure because it is culturally normative for women to move to their husband’s home after marriage and presumably adopt the household diet and lifestyle practices therein.

Missing data for any single outcome ranged from 0.2%–3% for common mental disorder, obesity, and hypertension to 24%–26% for diabetes and high cholesterol. To maintain the same analytic sample for each exposure-outcome model, we were thus forced to exclude 30% of the available participants because of missing data. We applied inverse probability weighting (IPW) to address potential bias arising from the exclusion of participants with missing data. IPW weights each observation by the inverse of the probability of having complete data to create a weighted pseudopopulation that resembles the full sample with respect to observed data [33]. We constructed the missing data IPW weights using a logistic model to predict the probability of having complete covariate data. The IPW model predictors were study site, age, sex, and education. We further accounted for the uneven number of adults in a single household contributing to the analysis using an IPW approach by creating a household weight that was the inverse of the household size. All analyses were weighted by a final weight that was the product of the missing data weight and the household weight and normalized to sum to the number of individuals with complete covariate data (7,522 participants). S1 Table shows missing data by covariate and descriptive analysis of participant characteristics in the total sample, unweighted analytic sample, and weighted analytic sample.

Adjusted models included the age, sex, education, marital status, religion, and study site of the index participant whose outcome was being modeled. Supplementary tables include results from unadjusted models. Data from Madhya Pradesh were excluded from the analysis of common mental disorder because less than 0.01% of respondents reported symptoms consistent with the common mental disorder definition. All analyses were model-based and accounted for data correlation arising from sampling multiple individuals in the same household and in the same cluster through generalized estimating equations [34]. Data management and recoding were performed using STATA 13 and 14 (College Station, Texas, United States), and statistical analyses were performed using SAS 9.4 (Cary, North Carolina, US) statistical software.

## Results

Table 1 shows the household- and individual-level characteristics of the weighted analytic sample. A total of 2,574 households with 7,522 individuals were analyzed. On average, we observed 2.9 individuals per household (range: 2 to 11 individuals). The mean age of participants was 39 years (range: 18 to 96 years), 46% were men, and mean years of schooling of participants was 6 years. The majority of participants were married (78%) and Hindu (94%). While 43% of individuals had at least 1 chronic condition, this proportion varied from 37% in Mashobra to 50% in Gujarat. The least common chronic condition was high cholesterol (6%), and the most common condition was hypertension (23%).

**Table 1:** Characteristics of the analytic sample.

	Unweighted sample size				
Characteristic	Total sample	Dhar, Madhya Pradesh	Junagadh, Gujarat	Pondicherry, Pudicherry	Mashobra, Himachal Pradesh
**Number of villages**	45	12	12	12	9
**Number of households**	2,574	827	799	484	464
**Number of individuals**	7,522	2,714	2,297	1,222	1289
**Household level**	Weighted mean or percent (standard error)				
Household size, mean	2.92 (0.2)	2.7 (0.0)	2.5 (0.0)	3.8 (0.1)	3.0 (0.1)
Percent of men in household, mean	46.4 (0.4)	50.9 (0.6)	48.4 (0.7)	42.2 (1.0)	39.5 (1.2)
Mean age in household, mean	38.7 (0.2)	33.8 (0.2)	39.8 (0.3)	41.1 (0.5)	43.3 (0.5)
**Individual level**					
*Demographic characteristics*					
Age, years, mean	39.2 (0.2)	33.8 (0.2)	39.9 (0.3)	40.8 (0.4)	43.2 (0.5)
Men, %	46.1 (0.4)	51.3 (0.5)	48.6 (0.7)	42.9 (1.0)	40.0 (1.2)
Education, years, mean	6.3 (0.1)	3.0 (0.1)	6.8 (0.1)	7.9 (0.2)	8.0 (0.2)
Married, %	78.7 (0.6)	84.6 (0.8)	78.7 (1.0)	75.1 (1.3)	75.3 (1.3)
Hindu, %	94.3 (0.4)	99.4 (0.2)	83.2 (1.3)	96.8 (0.8)	99.3 (0.3)
*Objectively assessed chronic condition status*					
Any chronic condition, %	43.7 (0.7)	38.5 (1.1)	50.2 (1.1)	47.8 (1.6)	36.6 (1.5)
Diabetes, %	11.0 (0.4)	13.6 (0.8)	9.1 (0.7)	15.5 (1.2)	4.1 (0.6)
Common mental disorder, %	13.2 (0.5)	0.1 (0.1)	24.2 (1.1)	17.1 (1.2)	10.9 (1.0)
Hypertension, %	23.3 (0.6)	24.2 (0.9)	25.7 (1.0)	21.3 (1.3)	21.4 (1.2)
Obesity, %	8.0 (0.4)	3.1 (0.4)	12.1 (0.8)	11.1 (1.0)	4.8 (0.6)
High cholesterol, %	5.5 (0.3)	3.9 (0.4)	4.6 (0.5)	6.8 (0.8)	7.1 (0.8)

Abbreviations: CI, confidence interval.

Data are from 7,522 adults in 2,574 households in the DISHA study. Chronic conditions were defined as follows: diabetes, fasting plasma glucose ≥ 126 mg/dL or taking medication; common mental disorder, General Health Questionnaire score ≥ 12; hypertension, blood pressure ≥ 140/90 mmHg or taking medication; obesity, body mass index ≥ 30 kg/m^2^; and high cholesterol, total blood cholesterol ≥ 240 mg/dL or taking medication. The sums of the weights in the analytic sample by site were 2,026 (Dhar), 2,039 (Junagadh), 1,957 (Pondicherry), and 1,500 (Mashobra).

Table 2 summarizes the results from separate adjusted logistic regression models estimating the odds ratio for having a given chronic condition if living with an individual with that same condition (Table 2, diagonal cells) and living with an individual with a different chronic condition (Table 2, off-diagonal cells); see S2 Table for unadjusted associations. Those who resided with another individual with any chronic condition had 29% higher adjusted relative odds of having any chronic condition themselves (adjusted odds ratio [aOR] = 1.29; 95% confidence interval [95% CI] 1.10–1.50). In general, the strongest relationships were observed in the same-condition models; positive associations were observed for diabetes (aOR = 1.60; 95% CI 1.23–2.07), common mental disorder (aOR = 2.69; 95% CI 2.12–3.42), and obesity (aOR = 1.82; 95% CI 1.33–2.50). With respect to differing conditions, the only statistically significant relationship was that between living with someone with hypertension and obesity status (aOR = 1.24; 95% CI 1.02–1.53).

**Table 2:** Adjusted association between living with someone with a given chronic condition and having that same or another chronic condition (*n* = 7,572).

Chronic condition present in at least 1 other adult in household (exposure)	Adjusted relative odds of chronic condition in any adult household member											
	Any chronic condition		Diabetes		Common mental disorder		Hypertension		Obesity		High cholesterol	
	OR (95% CI)	*p*	OR (95% CI)	*p*	OR (95% CI)	*p*	OR (95% CI)	*p*	OR (95% CI)	*p*	OR (95% CI)	*p*
Any chronic condition	1.29 (1.10–1.50)	<0.01	1.20 (0.98–1.46)	0.07	1.61 (1.31–1.98)	<0.01	1.18 (1.01–1.37)	0.04	1.18 (0.94–1.48)	0.16	1.19 (0.92–1.53)	0.18
Diabetes	1.21 (1.04–1.40)	0.01	1.60 (1.23–2.07)	<0.01	0.98 (0.77–1.25)	0.89	1.17 (1.00–1.37)	0.05	1.24 (0.97–1.59)	0.08	1.23 (0.91–1.65)	0.18
Common mental disorder	1.48 (1.26–1.75)	<0.01	0.90 (0.70–1.15)	0.39	2.69 (2.12–3.42)	<.01	1.11 (0.94–1.33)	0.23	1.00 (0.78–1.27)	0.99	0.96 (0.71–1.30)	0.79
Hypertension	1.17 (1.04–1.33)	0.01	1.13 (0.95–1.35)	0.16	1.08 (0.91–1.29)	0.37	1.18 (0.99–1.40)	0.06	1.24 (1.02–1.52)	0.03	0.96 (0.76–1.22)	0.75
Obesity	1.19 (1.01–1.42)	0.04	1.12 (0.89–1.42)	0.33	1.01 (0.79–1.30)	0.93	1.18 (0.98–1.41)	0.08	1.82 (1.33–2.50)	<0.01	1.23 (0.91–1.68)	0.18
High cholesterol	1.21 (1.00–1.46)	0.05	1.27 (0.97–1.67)	0.09	1.12 (0.82–1.53)	0.47	1.01 (0.82–1.24)	0.91	1.34 (0.99–1.81)	0.06	1.41 (0.90–2.20)	0.13

Notes: Data from 7,522 adults residing in 2,574 households contributed to each model; the mean number of adults per household was 2.9. Chronic conditions were defined as follows: diabetes, fasting plasma glucose ≥ 126 mg/dL or taking medication; common mental disorder, General Health Questionnaire score ≥ 12; hypertension, blood pressure ≥ 140/90 mmHg or taking medication; obesity, body mass index ≥ 30 kg/m^2^; and high cholesterol, total blood cholesterol ≥ 240 mg/dL or taking medication. The diagonal cells show the odds ratios for the same condition, and the off-diagonal cells show the odds ratios for differing conditions between the participant and household disease status. Models were adjusted for the index participant’s age, sex, marital status, education, and study site. Data from Madhya Pradesh were excluded from the common mental disorder analyses.

**Fig 1:**
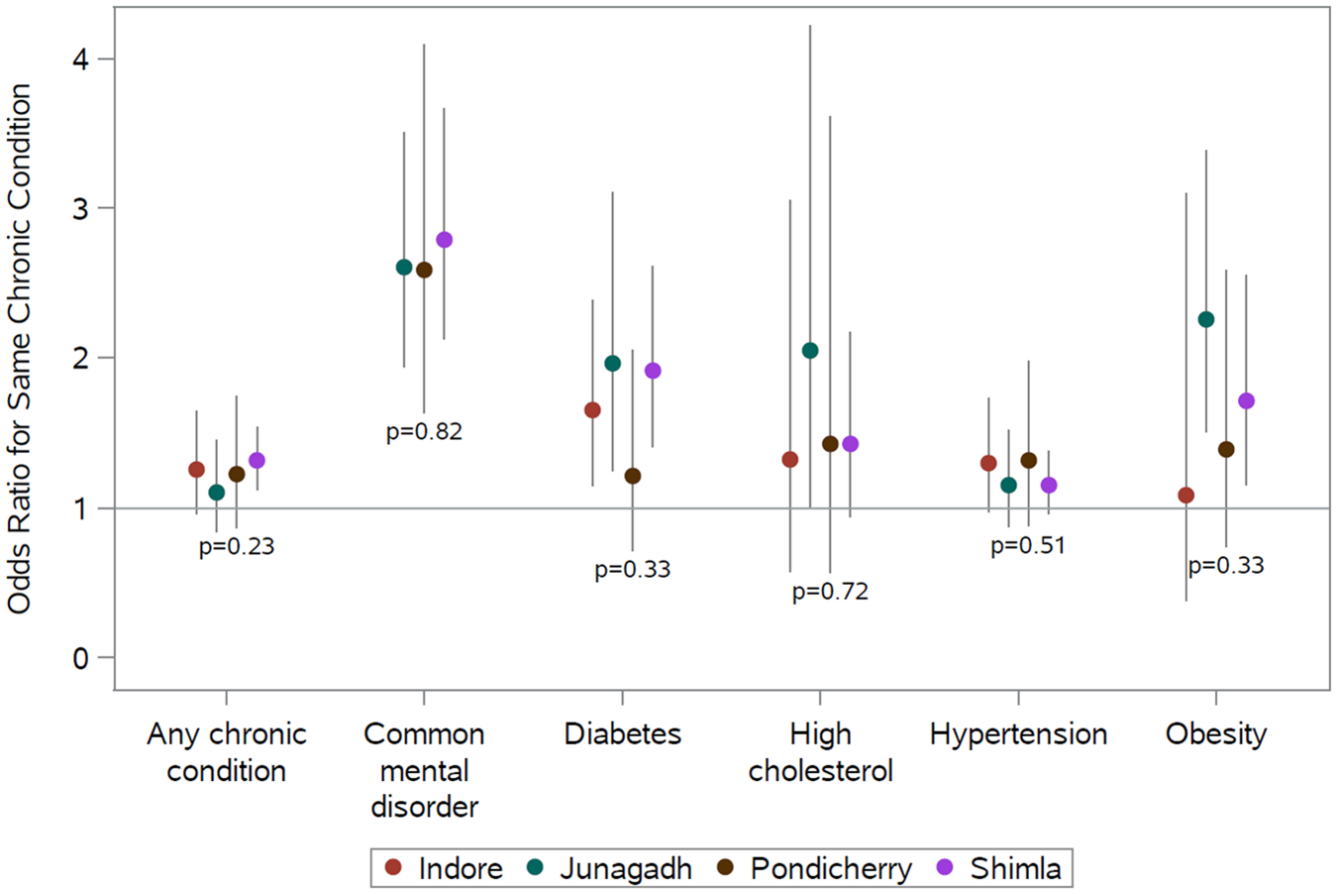
Site-specific adjusted relative odds (95% confidence interval) of having a chronic condition if any other member of the household has that same chronic condition (reference: no other member of the household has that same condition) and test for interaction between sites. Site-specific associations were computed by including an interaction term between the site and the exposure condition. The *P* values shown are from generalized score tests for Type III contrasts for the site x exposure interaction term. The horizontal line marks the null value. Madhya Pradesh data were excluded from the common mental disorder analysis because of poor performance of the survey tool. Chronic conditions were defined as follows: diabetes (prior diagnosis, fasting plasma glucose ≥ 126 mg/dL, or taking medication); common mental disorder (General Health Questionnaire score ≥ 12); hypertension (prior diagnosis, blood pressure ≥ 140/90 mmHg, or taking medication); obesity (body mass index ≥ 30 kg/m^2^); and high cholesterol (prior diagnosis, total blood cholesterol ≥ 240 mg/dL, or taking medication). See S3 Table for these data in table form.

Fig 1 shows the adjusted association between living with someone with a given chronic condition and having that same chronic condition by study site in the full analytic sample. See S3 Table for point estimates, CIs, and interaction tests in table form. There were no statistically significant differences between sites, and point estimates of the odds ratios indicate positive associations for concordant conditions among coresiding household members at all sites. Point estimates of odds ratios for any chronic condition, depression, and hypertension were very comparable across sites. Although not statistically distinguishable, point estimates of odds ratios for diabetes, high cholesterol, and obesity were more variable than those observed for any chronic condition, depression, or hypertension.

Table 3 shows adjusted associations between the chronic condition status of parents and their adult children (sample restricted to parent-child dyads); see S4 Table for unadjusted associations. Adults coresiding with a parent who had a common mental disorder (aOR = 2.20; 95% CI 1.28–3.77), hypertension (aOR = 1.58; 95% CI 1.15–2.16), obesity (aOR = 4.99; 95% CI 2.71–9.20), or high cholesterol (aOR = 2.57; 95% CI 1.15–5.73) were more likely to have the same respective condition. We found no statistically significant association between parents and their adult children for any chronic condition or diabetes. The only statistically significant relationships between differing chronic conditions were those between parental high cholesterol and adult child diabetes (aOR = 2.02; 95% CI 1.08–3.78) and between parental diabetes and adult child hypertension (aOR = 1.97; 95% CI 1.28–3.02).

**Table 3:** Adjusted association between living with a parent with a given chronic condition and having that same or another chronic condition (*n* = 1,660).

Parents’ chronic condition status (exposure)	Adjusted relative odds of chronic condition in adult child											
	Any chronic condition		Diabetes		Common mental disorder		Hypertension		Obesity		High cholesterol	
	OR (95% CI)	*p*	OR (95% CI)	*p*	OR (95% CI)	*p*	OR (95% CI)	*p*	OR (95% CI)	*p*	OR (95% CI)	*p*
Any chronic condition	1.15 (0.88–1.51)	0.31	0.77 (0.49–1.19)	0.24	1.14 (0.62–2.11)	0.67	1.49 (1.07–2.09)	0.02	1.35 (0.70–2.64)	0.37	1.56 (0.77–3.17)	0.22
Diabetes	1.35 (0.93–1.97)	0.12	0.58 (0.29–1.16)	0.12	1.20 (0.56–2.60)	0.64	1.97 (1.28–3.02)	<0.01	1.46 (0.68–3.14)	0.34	1.18 (0.50–2.82)	0.70
Common mental disorder	1.10 (0.77–1.59)	0.59	1.00 (0.49–2.05)	1.00	2.20 (1.28–3.77)	<0.01	0.88 (0.53–1.48)	0.64	0.72 (0.33–1.58)	0.42	0.52 (0.20–1.32)	0.17
Hypertension	1.24 (0.97–1.60)	0.09	0.62 (0.40–0.94)	0.03	1.09 (0.66–1.80)	0.74	1.58 (1.15–2.16)	<0.01	1.50 (0.82–2.76)	0.19	1.81 (0.90–3.67)	0.10
Obesity	1.62 (1.13–2.31)	<0.01	0.64 (0.32–1.30)	0.22	1.25 (0.65–2.37)	0.50	1.56 (0.99–2.44)	0.05	4.99 (2.71–9.20)	<.01	1.77 (0.70–4.48)	0.22
High cholesterol	1.31 (0.89–1.95)	0.18	2.02 (1.08–3.78)	0.03	1.12 (0.53–2.34)	0.77	1.16 (0.68–1.98)	0.58	0.89 (0.31–2.54)	0.83	2.57 (1.15–5.73)	0.02

Notes: Data from 1,660 parents and coresiding adult children in 1,199 households contributed to each model. Chronic conditions were defined as follows: diabetes, fasting plasma glucose ≥ 126 mg/dL or taking medication; common mental disorder, General Health Questionnaire score ≥ 12; hypertension, blood pressure ≥ 140/90 mmHg or taking medication; obesity, body mass index ≥ 30 kg/m^2^; and high cholesterol, total blood cholesterol ≥ 240 mg/dL or taking medication. Parental chronic condition status was coded as positive if 1 or both parents had the condition. The diagonal cells show the odds ratios for the same condition, and the off-diagonal cells show the odds ratios for differing conditions between the parent and the adult child. Models were adjusted for age, sex, education, marital status, religion, and site of the adult child. Data from Madhya Pradesh were excluded from the common mental disorder analyses.

Table 4 shows adjusted associations between chronic condition status of spousal dyads; see S5 Table for unadjusted associations. A woman had 44% higher adjusted relative odds of having a chronic condition if her husband had a chronic condition (aOR = 1.44; 95% CI 1.14–1.82). Similar to the analyses above, the strongest relationships were seen for the same condition. Concordant diabetes (aOR = 2.28; 95% CI 1.52–3.42) and common mental disorder (aOR = 3.01; 95% CI 2.01–4.52) status in husbands and wives were the only statistically significant associations among spouses.

**Table 4:** Adjusted association between living with a spouse with a given chronic condition and having that same or another chronic condition (*n* = 1,598).

Husband’s chronic condition status	Adjusted relative odds of chronic condition in wives											
	Any chronic condition		Diabetes		Common mental disorder		Hypertension		Obesity		High cholesterol	
	OR (95% CI)	*p*	OR (95% CI)	*p*	OR (95% CI)	*p*	OR (95% CI)	*p*	OR (95% CI)	*p*	OR (95% CI)	*p*
Any chronic condition	1.44 (1.14–1.82)	<0.01	1.59 (1.11–2.30)	0.01	1.50 (1.06–2.12)	0.02	1.40 (1.07–1.84)	0.01	1.17 (0.84–1.64)	0.35	1.71 (1.06–2.75)	0.03
Diabetes	1.21 (0.87–1.68)	0.26	2.28 (1.52–3.42)	<0.01	0.86 (0.54–1.37)	0.53	1.19 (0.83–1.70)	0.34	1.47 (0.98–2.20)	0.06	1.41 (0.78–2.54)	0.26
Common mental disorder	2.58 (1.63–4.09)	<0.01	0.87 (0.41–1.86)	0.73	3.01 (2.01–4.52)	<0.01	1.28 (0.79–2.06)	0.32	0.98 (0.59–1.64)	0.94	1.61 (0.76–3.41)	0.22
Hypertension	1.21 (0.94–1.55)	0.13	1.51 (1.05–2.17)	0.03	0.98 (0.68–1.42)	0.92	1.20 (0.91–1.59)	0.19	0.86 (0.61–1.20)	0.38	1.18 (0.73–1.90)	0.51
Obesity	0.82 (0.49–1.38)	0.45	1.29 (0.61–2.71)	0.50	0.64 (0.27–1.51)	0.31	1.23 (0.69–2.18)	0.49	1.60 (0.90–2.85)	0.11	0.82 (0.25–2.70)	0.75
High cholesterol	1.06 (0.66–1.69)	0.81	1.29 (0.69–2.44)	0.43	1.09 (0.58–2.02)	0.79	0.95 (0.56–1.63)	0.86	1.72 (1.00–2.97)	0.05	0.77 (0.31–1.91)	0.57

Notes: Data from 1,598 spousal dyads contributed to each model. Chronic conditions were defined as follows: diabetes, fasting plasma glucose ≥ 126 mg/dL or taking medication; common mental disorder, General Health Questionnaire score ≥ 12; hypertension, blood pressure ≥ 140/90 mmHg or taking medication; obesity, body mass index ≥ 30 kg/m^2^; and high cholesterol, total blood cholesterol ≥ 240 mg/dL or taking medication. The diagonal cells show the odds ratios for the same condition, and the off-diagonal cells show the odds ratios for differing conditions between the husband and wife. Models were adjusted for wife’s age and sex and the mean of years of husband’s and wife’s education, religion, and study site. Data from Madhya Pradesh were excluded from the common mental disorder analyses.

## Discussion

To our knowledge, this is the first study examining the relationship of 5 prevalent chronic conditions—hypertension, diabetes, obesity, common mental disorder, and high cholesterol—among coresiding adults in India. Irrespective of familial relationship, adults who resided with another adult with any chronic condition had 29% higher adjusted odds of having 1 or more chronic conditions themselves. For all of the 5 specific conditions examined, we consistently observed that adults tended to have the same chronic condition as a coresiding household member (e.g., living with someone who is obese was associated with 82% higher relative odds of obesity). Among all household members, parent-adult child dyads, and spousal dyads, common mental disorder was twice to thrice as high among individuals residing with someone with a common mental disorder. Other salient findings included the strong concordance of diabetes status among husbands and wives and concordance of obesity status among parents and their adult children. Across different disease phenotypes, we observed weaker and few statistically significant associations among household members.

In India, the past 20 years of unprecedented economic growth [35] has coincided with an increase in healthy life expectancy in men and women by 6 and 9 years, respectively [36]. Chronic diseases, however, threaten continued progress in this arena. There is a great need to reorient the existing health system in India to address the rise of chronic conditions [5,37]. The bulk of familial concordance studies have been conducted to understand the genetic influences of parents on young or adolescent children or environmental influences on health among spouses [7]. In India [4] and other LMICs [25] where extended family households are still intact, it may be particularly relevant and effective to engage the full household—irrespective of genetic ties or marital connections—in the prevention and management of chronic conditions. At the most superficial level, family history of chronic disease can be used for risk stratification [38].

Our findings are largely consistent with prior literature examining the relationship of metabolic outcomes among spouses [10–12,39] and between parents and adolescent children [11–13] within nuclear families. Specifically, the concordance of chronic condition status among spouses in our study was remarkably similar to findings published in a systematic review, reporting odds ratios between 1.2 and 1.6 for hypertension, 1.1 to 1.8 for diabetes, and 1.3 to 1.7 for obesity [6]. Additionally, there is robust concordance in these metabolic outcomes as a package (i.e., metabolic syndrome) among spouses [10–12]. Regarding phenotypic similarities between parents and their adult children, our results tended to demonstrate more statistically robust relationships compared with prior studies [11,12], likely due to a larger sample size and our restriction to adult children (versus adolescents and younger children). Previous analyses of chronic disease-related traits—such as continuously measured blood pressure and body mass index (BMI)—have also found that these traits are related in parent-child dyads and cluster within the household [8,9,13,40,41]. Across these previous studies and ours, generally, the correlation of obesity/BMI among family members was stronger than the correlation of hypertension/blood pressure. Comparable to what we found for common mental disorder, a single study examining concordance of multiple diseases among married couples estimated an aOR of 2.1 for depression and also reported that depression was the most concordant condition of the many outcomes examined in the study [39].

Although we did not directly or quantitatively examine contributing pathways in this study, qualitative comparisons of coefficients across models may provide a preliminary understanding for future investigation. First, odds ratios for chronic condition concordance adjusted for sociodemographic information (such as educational level and site) were generally attenuated compared with unadjusted odds ratios; the greatest attenuation after adjustment was observed in the spousal concordance analysis (Table 4 versus S5 Table). To the extent that sociodemographic background proxies living conditions, this suggests that shared living conditions are relevant to determining spousal chronic condition concordance. Second, common mental disorder was the only condition that was highly and statistically significantly concordant in models including all household members, models restricted to dyads of parents and their adult children, and models restricted to dyads of spouses, implying a potential affective contagion that affects members of a household irrespective of familial relationship. Third, concordance in hypertension and high cholesterol were only observed in parent-adult child dyads, implying a potential genetic component for observed household concordance in those conditions. Fourth, household concordance in diabetes was not observed in parent-adult child dyads, and concordance in obesity was not statistically significant among spouses. Additionally, these findings run contrary to prior findings that metabolic syndrome is correlated in parents and their children [11,12] and that weight status is correlated among spouses [7,42]. The results raise the question of whether diabetes has a stronger environmental component and obesity has a stronger genetic component in this setting. Fifth, site-specific associations indicate a general tendency towards mild to moderate concordance of the 5 chronic conditions examined here, but diabetes, high cholesterol, and obesity odds ratios varied more than other conditions. Perhaps these conditions are more impacted by extrahousehold factors. Finally, correspondence across differing phenotypes was weaker than concordance of the same condition—even among spouses—possibly suggesting specificity of mechanisms contributing to each of the disease outcomes under study. Further data are needed to determine the robustness of each of these observations.

The DISHA study provided a geographically and socioculturally diverse study population for our secondary data analysis of the relationships between chronic conditions among members of the same household, yielding a strong foundation for generalizing findings across India and possibly other LMICs where extended family households remain common [25]. A strength of this data source was our ability to objectively characterize 5 chronic conditions and subsequently examine associations among coresiding adults in a large sample of Indian households using very recent data. Not only did we report the concordance of the same chronic condition among household members, but we also reported the correspondence between different chronic conditions. Although this approach required making several comparisons, these comparisons addressed our predefined research question regarding potential heightened risk for chronic conditions across differing phenotypes, and thus, we do not adjust for multiple comparisons in the analysis [43]. Moreover, our comprehensive analysis of these relationships among all household members, parent-adult child dyads, and spousal dyads separately provides data on the concordance and correspondence between chronic conditions in genetically related and unrelated adults who share a common living environment. Site-specific analysis assures us that the findings were consistent across the heterogeneous districts.

DISHA, however, was designed not to examine family-level associations in outcomes but rather to detect a 2 mmHg mean difference in systolic blood pressure between intervention and control villages. Thus, we had relatively small samples in the dyadic analysis, and we interpret tests of statistical significance and any qualitative differences between the associations observed for spousal versus parent-child dyads with caution. We also lacked sufficient sample size to examine associations in other potential familial relationships of interest (e.g., daughters-in-law and parents-in-law or disaggregated mother-child from father-child). In addition, 25% of the DISHA participants were missing fasting blood samples, which led to a high proportion of missing data. We addressed missing data using inverse probability weighting, which may negatively impact statistical precision. Finally, we were unable to investigate the development of new chronic conditions in household members—which could provide insight into the etiology of household clustering of disease—because of the cross-sectional nature of the analysis.

Our results provide preliminary evidence that targeting households in which 1 adult has a chronic condition may be an effective way to identify other individuals with chronic conditions and potentially prevent emerging chronic conditions in India, as has been done among spouses elsewhere [44,45]. Moreover, studying the incidence of chronic disease within household members may provide new insight regarding mechanisms relevant to primordial prevention of chronic disease risk factors. It is thus critical to substantiate these early findings in ongoing prospective cohort studies to better understand the mechanisms through which coresiding household members develop chronic conditions over time. Elucidating such mechanisms can assist with designing novel interventions that cater to the needs of households across the socioeconomic spectrum in both urban and rural settings. The design and subsequent rigorous evaluation of such interventions will contribute to generate the evidence base needed to combat the rise of chronic conditions in India.

## Supporting information

S1 Table Missing data by covariate and participant characteristics in the total sample, unweighted analytic sample, and weighted analytic sample. (DOCX)

S2 Table Unadjusted association between living with someone with a given chronic condition and having that same or another chronic condition. (DOCX)

S3 Table Site-specific adjusted relative odds (95% confidence interval) of having a chronic condition if any other member of the household has that same chronic condition (reference: no other member of the household has that same condition) and tests for interaction by study site. (DOCX)

S4 Table Unadjusted association between living with a parent with a given chronic condition and having that same or another chronic condition. (DOCX)

S5 Table Unadjusted association between living with a spouse with a given chronic condition and having that same or another chronic condition. (DOCX)

S1 Text Strengthening the Reporting of Observational Studies in Epidemiology (STROBE) statement—Checklist of items that should be included in reports of observational studies. (DOC)

S2 Text Development of the statistical analysis plan. (DOCX)

## Abbreviations

aOR: adjusted odds ratio
BMI: body mass index
CI: confidence interval
DISHA: Diet and Lifestyle Interventions for Hypertension Risk Reduction through Anganwadi Workers and Accredited Social Health Activists
HICs: high-income countries
LMICs: low- and middle-income countries
STROBE: Strengthening the Reporting of Observational Studies in Epidemiology
