# Supplementary material for: Chronic disease concordance within Indian households: A cross-sectional study
Source: PLoS Med. 2017 Sep 29;14(9):e1002395. doi: 10.1371/journal.pmed.1002395 (PMC5621663; doi:10.1371/journal.pmed.1002395)
Supplement: S1 Table — (DOCX) [file pmed.1002395.s001.docx]

S1 Table. Missing data by covariate and participant characteristics in the total sample, unweighted analytic sample, and weighted analytic sample.

|  |  |  | Prevalence (SE)* in the sample | | |
| --- | --- | --- | --- | --- | --- |
|  | % missing covariate |  |  | Sample with complete data of interest | |
| Variable Name |  |  | Total Sample | Weighted | Unweighted |
| Indore (binary) | 0 |  | 26.6 (0.7) | 26.9 (0.8) | 32.6 (0.9) |
| Junagadh (binary) | 0 |  | 26.9 (0.7) | 27.1 (0.9) | 31.5 (0.9) |
| Shimla (binary) | 0 |  | 19.8 (0.7) | 20.0 (0.8) | 18.5 (0.8) |
| Pondicherry (binary) | 0 |  | 26.7 (0.7) | 26.0 (1.0) | 17.3 (0.7) |
| Age (years) | 0 |  | 39.2 (0.2) | 39.2 (0.2) | 38.8 (0.2) |
| Male (binary) | 0 |  | 45.7 (0.3) | 46.1 (0.4) | 46.4 (0.4) |
| Education (years) | 0 |  | 6.3 (0.1) | 6.3 (0.1) | 5.9 (0.1) |
| Married (binary) | 0 |  | 78.3 (0.5) | 78.7 (0.6) | 79.6 (0.5) |
| Hindu (binary) | 0 |  | 94.3 (0.4) | 94.3 (0.4) | 93.6 (0.5) |
| Any chronic condition (binary) | 27.0 |  | 44.3 (0.6) | 43.7 (0.7) | 43.6 (0.6) |
| Diabetes (binary) | 25.5 |  | 12.1 (0.4) | 10.8 (0.4) | 10.8 (0.4) |
| Depression (binary) | 0.2 |  | 13.4 (0.4) | 13.2 (0.5) | 12.7 (0.5) |
| Hypertension (binary) | 2.5 |  | 23.2 (0.5) | 23.2 (0.6) | 23.7 (0.5) |
| Obesity (binary) | 3.0 |  | 7.4 (0.3) | 7.9 (0.4) | 7.7 (0.4) |
| High Cholesterol (binary) | 23.9 |  | 5.5 (0.3) | 5.5 (0.3) | 5.3 (0.3) |

*Mean (SE) is shown for age and education

Notes: The total sample was weighted by the household weight alone, while the weighted sample is weighted by the final weight (combining missing data and the household size). The weighted sample was used in the analysis.
