# Supplementary material for: Chronic disease concordance within Indian households: A cross-sectional study
Source: PLoS Med. 2017 Sep 29;14(9):e1002395. doi: 10.1371/journal.pmed.1002395 (PMC5621663; doi:10.1371/journal.pmed.1002395)
Supplement: S2 Table — (DOCX) [file pmed.1002395.s002.docx]

S2 Table. Unadjusted association between living with someone with a given chronic condition and having that same or another chronic condition (n=7572)

|  | **Unadjusted relative odds of chronic condition in any adult household member** | | | | | | | | | | | |
| --- | --- | --- | --- | --- | --- | --- | --- | --- | --- | --- | --- | --- |
|  | Any chronic condition | | Diabetes | | Common mental disorder | | Hypertension | | Obesity | | High cholesterol | |
| Chronic condition present in at least one other adult in household  (exposure) | OR (95% CI) | p | OR (95% CI) | p | OR (95% CI) | p | OR (95% CI) | p | OR (95% CI) | p | OR (95% CI) | p |
| Any chronic condition | 1.34 (1.15-1.55) | <.01 | 1.31 (1.08-1.59) | <.01 | 1.63 (1.34-1.99) | <.01 | 1.26 (1.09-1.46) | <.01 | 1.29 (1.04-1.61) | 0.02 | 1.21 (0.94-1.55) | 0.14 |
| Diabetes | 1.20 (1.04-1.37) | 0.01 | 1.91 (1.49-2.46) | <.01 | 0.99 (0.79-1.23) | 0.90 | 1.19 (1.03-1.39) | 0.02 | 1.31 (1.03-1.65) | 0.03 | 1.18 (0.88-1.58) | 0.28 |
| Common mental disorder | 1.45 (1.24-1.68) | <.01 | 0.97 (0.78-1.22) | 0.82 | 2.69 (2.15-3.37) | <.01 | 1.15 (0.98-1.34) | 0.09 | 1.02 (0.81-1.28) | 0.87 | 0.91 (0.68-1.22) | 0.52 |
| Hypertension | 1.19 (1.06-1.34) | <.01 | 1.23 (1.03-1.46) | 0.02 | 1.13 (0.96-1.33) | 0.15 | 1.24 (1.05-1.46) | <.01 | 1.33 (1.09-1.61) | <.01 | 0.98 (0.78-1.23) | 0.86 |
| Obesity | 1.32 (1.13-1.54) | <.01 | 1.27 (1.01-1.59) | 0.04 | 0.95 (0.77-1.19) | 0.68 | 1.30 (1.10-1.53) | <.01 | 1.99 (1.46-2.70) | <.01 | 1.33 (0.99-1.78) | 0.06 |
| High cholesterol | 1.15 (0.96-1.37) | 0.13 | 1.26 (0.95-1.65) | 0.10 | 1.00 (0.76-1.32) | 1.00 | 1.02 (0.84-1.25) | 0.82 | 1.35 (1.00-1.81) | 0.05 | 1.49 (0.96-2.31) | 0.07 |

Notes: Data from 7,522 adults residing in 2574 households contributed to each model; the mean number of adults per household was 2.9. Chronic conditions were defined as follows: diabetes, fasting plasma glucose≥126 mg/dL or taking medication; common mental disorder, General Health Questionnaire score ≥ 12; hypertension, blood pressure ≥ 140/90 mmHg or taking medication; obesity, body mass index ≥30 kg/m^2^; high cholesterol, total blood cholesterol ≥ 240 mg/dL or taking medication. The diagonal cells show the odds ratios for the same condition and the off-diagonal cells show the odds ratios for differing conditions between the participant and household disease status. Data from Madhya Pradesh were excluded from the common mental disorder analyses.
