# Supplementary material for: Chronic disease concordance within Indian households: A cross-sectional study
Source: PLoS Med. 2017 Sep 29;14(9):e1002395. doi: 10.1371/journal.pmed.1002395 (PMC5621663; doi:10.1371/journal.pmed.1002395)
Supplement: S3 Table — (DOCX) [file pmed.1002395.s003.docx]

S3 Table. Site-specific adjusted relative odds (95% confidence interval) of having a chronic condition if any other member of the household has that same chronic condition (reference: no other member of the household has that same condition) and tests for interaction by study site

| Chronic Condition | Site | Adjusted Odds Ratio (aOR) | Lower Confidence Limit of the aOR | Upper Confidence Limit of the aOR | *p-*value for site OR | *p*-value for site inter-action* |
| --- | --- | --- | --- | --- | --- | --- |
| Any chronic condition | Dhar | 1.2555 | 0.9590 | 1.6438 | 0.098 | 0.2310 |
|  | Junagadh | 1.1025 | 0.8398 | 1.4476 | 0.482 |  |
|  | Pondicherry | 1.2261 | 0.8610 | 1.7461 | 0.258 |  |
|  | Mashobra | 1.3149 | 1.1220 | 1.5410 | <.001 |  |
| Common mental disorder | Junagadh | 2.6074 | 1.9412 | 3.5022 | <.001 | 0.8211 |
|  | Pondicherry | 2.5838 | 1.6315 | 4.0919 | <.001 |  |
|  | Mashobra | 2.7875 | 2.1221 | 3.6615 | <.001 |  |
| Diabetes | Dhar | 1.6521 | 1.1443 | 2.3852 | 0.007 | 0.3307 |
|  | Junagadh | 1.9658 | 1.2453 | 3.1031 | 0.004 |  |
|  | Pondicherry | 1.2088 | 0.7110 | 2.0552 | 0.484 |  |
|  | Mashobra | 1.9163 | 1.4069 | 2.6101 | <.001 |  |
| High cholesterol | Dhar | 1.3219 | 0.5728 | 3.0507 | 0.513 | 0.7174 |
|  | Junagadh | 2.0486 | 0.9948 | 4.2185 | 0.052 |  |
|  | Pondicherry | 1.4259 | 0.5629 | 3.6122 | 0.454 |  |
|  | Mashobra | 1.4227 | 0.9335 | 2.1684 | 0.101 |  |
| Hypertension | Dhar | 1.2978 | 0.9741 | 1.7291 | 0.075 | 0.5071 |
|  | Junagadh | 1.1487 | 0.8681 | 1.5200 | 0.332 |  |
|  | Pondicherry | 1.3169 | 0.8773 | 1.9768 | 0.184 |  |
|  | Mashobra | 1.1507 | 0.9606 | 1.3784 | 0.128 |  |
| Obesity | Dhar | 1.0817 | 0.3776 | 3.0983 | 0.884 | 0.3328 |
|  | Junagadh | 2.2563 | 1.5029 | 3.3874 | <.001 |  |
|  | Pondicherry | 1.3860 | 0.7428 | 2.5863 | 0.305 |  |
|  | Mashobra | 1.7108 | 1.1479 | 2.5498 | 0.008 |  |

*Statistical differences in the odds ratio for disease concordance was tested using an interaction term between the chronic condition exposure and the study site (specified as a 4-level categorical variable). Statistical significance of the interaction term using generalized score tests for Type III contrasts.
