# Supplementary material for: Chronic disease concordance within Indian households: A cross-sectional study
Source: PLoS Med. 2017 Sep 29;14(9):e1002395. doi: 10.1371/journal.pmed.1002395 (PMC5621663; doi:10.1371/journal.pmed.1002395)
Supplement: S4 Table — (DOCX) [file pmed.1002395.s004.docx]

S4 Table. Unadjusted association between living with a parent with a given chronic condition and having that same or another chronic condition (n=1660)

|  | Relative odds of chronic condition in adult child | | | | | | | | | | | |
| --- | --- | --- | --- | --- | --- | --- | --- | --- | --- | --- | --- | --- |
|  | Any chronic condition | | Diabetes | | Common mental disorder | | Hypertension | | Obesity | | High cholesterol | |
| Parents’ chronic condition status  (exposure) | OR (95% CI) | p | OR (95% CI) | p | OR (95% CI) | p | OR (95% CI) | p | OR (95% CI) | p | OR (95% CI) | p |
| Any chronic condition | 1.27 (0.98-1.64) | 0.08 | 0.77 (0.50-1.18) | 0.23 | 1.25 (0.72-2.17) | 0.43 | 1.53 (1.10-2.13) | 0.01 | 1.75 (0.90-3.42) | 0.10 | 1.71 (0.86-3.40) | 0.13 |
| Diabetes | 1.55 (1.09-2.22) | 0.02 | 0.64 (0.33-1.24) | 0.19 | 1.22 (0.59-2.52) | 0.58 | 2.12 (1.39-3.23) | <.01 | 1.78 (0.86-3.69) | 0.12 | 1.59 (0.66-3.80) | 0.30 |
| Common mental disorder | 1.42 (1.02-1.99) | 0.04 | 1.29 (0.63-2.62) | 0.48 | 2.30 (1.41-3.76) | <.01 | 1.27 (0.79-2.04) | 0.32 | 0.89 (0.43-1.86) | 0.76 | 0.59 (0.25-1.38) | 0.23 |
| Hypertension | 1.43 (1.13-1.82) | <.01 | 0.67 (0.45-1.02) | 0.06 | 1.41 (0.87-2.30) | 0.17 | 1.77 (1.31-2.40) | <.01 | 1.80 (1.01-3.21) | 0.05 | 1.79 (0.95-3.37) | 0.07 |
| Obesity | 1.28 (0.92-1.79) | 0.15 | 0.50 (0.26-0.96) | 0.04 | 0.92 (0.50-1.69) | 0.78 | 1.15 (0.74-1.78) | 0.54 | 5.12 (2.81-9.34) | <.01 | 1.57 (0.64-3.87) | 0.33 |
| High cholesterol | 1.21 (0.82-1.80) | 0.34 | 1.61 (0.88-2.94) | 0.12 | 1.11 (0.53-2.31) | 0.79 | 0.99 (0.58-1.68) | 0.97 | 1.01 (0.35-2.94) | 0.99 | 2.74 (1.27-5.90) | <.01 |

Notes: Data from 1660 parents and co-residing adult children in 1199 households contributed to each model. Chronic conditions were defined as follows: diabetes, fasting plasma glucose≥126 mg/dL or taking medication; common mental disorder, General Health Questionnaire score ≥ 12; hypertension, blood pressure ≥ 140/90 mmHg or taking medication; obesity, body mass index ≥30 kg/m^2^; high cholesterol, total blood cholesterol ≥ 240 mg/dL or taking medication. Parental chronic conditions status was coded as positive if one or both parents had the condition. The diagonal cells show the odds ratios for the same condition and the off-diagonal cells show the odds ratios for differing conditions between the parent and the adult child. Data from Madhya Pradesh were excluded from the common mental disorder analyses.
