# Supplementary material for: Chronic disease concordance within Indian households: A cross-sectional study
Source: PLoS Med. 2017 Sep 29;14(9):e1002395. doi: 10.1371/journal.pmed.1002395 (PMC5621663; doi:10.1371/journal.pmed.1002395)
Supplement: S5 Table — (DOCX) [file pmed.1002395.s005.docx]

S5 Table. Unadjusted association between living with a spouse with a given chronic condition and having that same or another chronic condition (n= 1598)

|  | **Unadjusted relative odds of chronic condition in wives** | | | | | | | | | | | |
| --- | --- | --- | --- | --- | --- | --- | --- | --- | --- | --- | --- | --- |
|  | Any chronic condition | | Diabetes | | Common mental disorder | | Hypertension | | Obesity | | High cholesterol | |
| Husband’s chronic conditions status | OR (95% CI) | p | OR (95% CI) | p | OR (95% CI) | p | OR (95% CI) | p | OR (95% CI) | p | OR (95% CI) | p |
| Any chronic condition | 1.85 (1.49,2.30) | <.01 | 1.94 (1.35,2.78) | <.01 | 1.79 (1.30,2.45) | <.01 | 1.92 (1.49,2.48) | <.01 | 1.51 (1.10,2.07) | <.01 | 2.42 (1.53,3.84) | <.01 |
| Diabetes | 1.43 (1.06,1.95) | 0.02 | 2.92 (1.94,4.39) | <.01 | 0.97 (0.63,1.50) | 0.89 | 1.49 (1.06,2.10) | 0.02 | 1.72 (1.18,2.53) | <.01 | 1.71 (0.97,3.04) | 0.07 |
| Common mental disorder | 3.15 (2.07,4.78) | <.01 | 1.13 (0.61,2.10) | 0.69 | 4.22 (2.89,6.16) | <.01 | 1.83 (1.23,2.70) | <.01 | 1.01 (0.64,1.61) | 0.95 | 2.15 (1.17,3.94) | 0.01 |
| Hypertension | 1.54 (1.23,1.94) | <.01 | 1.83 (1.28,2.62) | <.01 | 1.15 (0.84,1.59) | 0.38 | 1.73 (1.33,2.25) | <.01 | 1.12 (0.81,1.53) | 0.50 | 1.81 (1.15,2.85) | 0.01 |
| Obesity | 0.94 (0.56,1.58) | 0.82 | 1.26 (0.59,2.71) | 0.55 | 0.65 (0.30,1.40) | 0.27 | 1.12 (0.62,2.01) | 0.71 | 2.04 (1.13,3.68) | 0.02 | 0.78 (0.26,2.32) | 0.65 |
| High cholesterol | 1.25 (0.80,1.93) | 0.32 | 1.42 (0.74,2.74) | 0.29 | 1.07 (0.61,1.87) | 0.81 | 1.05 (0.61,1.79) | 0.87 | 2.10 (1.27,3.50) | <.01 | 0.93 (0.37,2.33) | 0.88 |

Notes: Data from 1598 spousal dyads contributed to each model. Chronic conditions were defined as follows: diabetes, fasting plasma glucose≥126 mg/dL or taking medication; common mental disorder, General Health Questionnaire score ≥ 12; hypertension, blood pressure ≥ 140/90 mmHg or taking medication; obesity, body mass index ≥30 kg/m^2^; high cholesterol, total blood cholesterol ≥ 240 mg/dL or taking medication. The diagonal cells show the odds ratios for the same condition and the off-diagonal cells show the odds ratios for differing conditions between the husband and wife. Data from Madhya Pradesh were excluded from the common mental disorder analyses.
