# Supplementary material for: Chronic disease concordance within Indian households: A cross-sectional study
Source: PLoS Med. 2017 Sep 29;14(9):e1002395. doi: 10.1371/journal.pmed.1002395 (PMC5621663; doi:10.1371/journal.pmed.1002395)
Supplement: S1 Text — (DOC) [file pmed.1002395.s006.doc]

STROBE Statement—checklist of items that should be included in reports of observational studies

|  | Completion | Recommendation |
| --- | --- | --- |
| **Title and abstract** | Do Families Matter? A cross-sectional study of chronic disease concordance within Indian households  Please see the abstract for the text | (*a*) Indicate the study’s design with a commonly used term in the title or the abstract |
|  |  | (*b*) Provide in the abstract an informative and balanced summary of what was done and what was found |
| Introduction | | |
| Background/rationale | Understanding associations of shared and differing chronic conditions among all-co residing adults in households may shed light on new approaches to identify and treat chronic illness in low- and middle-income countries such as India. | Explain the scientific background and rationale for the investigation being reported |
| Objectives | The overarching goal of this study was to test the hypothesis that living with a household member who has a chronic condition—diabetes, common mental disorder, hypertension, and/or obesity—raises the risk of developing a chronic condition. | State specific objectives, including any prespecified hypotheses |
| Methods | | |
| Study design | We conducted a cross-sectional observational analysis of the baseline survey data from the DISHA intervention study. | Present key elements of study design early in the paper |
| Setting | DISHA is a community-based cluster randomized trial designed to test the effectiveness of a community health worker-led lifestyle behavior change on hypertension reduction. The baseline study was conducted in 2013-2014 to measure risk factors in 4 regionally and socioeconomically diverse districts located in Madhya Pradesh, Gujarat, Tamil Nadu, and Himachal Pradesh selected for the initial phase of the intervention. | Describe the setting, locations, and relevant dates, including periods of recruitment, exposure, follow-up, and data collection |
| Participants | Participants were selected using a multi-stage cluster sampling design stratified by district.  At the household level, all adults over the age of 18 were invited to participate in the survey. | (*a*) *Cohort study*—Give the eligibility criteria, and the sources and methods of selection of participants. Describe methods of follow-up  *Case-control study*—Give the eligibility criteria, and the sources and methods of case ascertainment and control selection. Give the rationale for the choice of cases and controls  ***Cross-sectional study*—Give the eligibility criteria, and the sources and methods of selection of participants** |
|  |  | (*b*) *Cohort study*—For matched studies, give matching criteria and number of exposed and unexposed  *Case-control study*—For matched studies, give matching criteria and the number of controls per case |
| Variables | We analyzed five prevalent chronic conditions: Diabetes (prior diagnosis by a physician, fasting plasma glucose≥126 mg/dL, or taking medication); common mental disorder (General Health Questionnaire score ≥ 12); hypertension (prior diagnosis by a physician, blood pressure ≥ 140/90 mmHg or taking medication); obesity (body mass index ≥30 kg/m^2^); and high cholesterol (prior diagnosis by a physician, total blood cholesterol ≥ 240 mg/dL or taking medication). We also created a composite binary variable indicating the presence of at least one of the five chronic conditions. | Clearly define all outcomes, exposures, predictors, potential confounders, and effect modifiers. Give diagnostic criteria, if applicable |
| Data sources/ measurement | Data collection took place at the participant’s home. Height was measured using a stadiometer with accuracy of 2mm (Seca), weight was measured using a digital weighing scale with accuracy of 100gm (Seca), and systolic and diastolic blood pressure was measured using an electronic blood pressure monitor (OMRON 7080). A 5 ml fasting blood sample was collected from participants reporting at least 9 hours of fasting. The sample was centrifuged in the field and the resulting serum and plasma samples were then transported to a central laboratory in New Delhi at the Indian Council of Medical Research for biochemical analysis and storage. Fasting plasma glucose was assessed using the Enzymatic Colorimetric Assay method. The General Health Questionnaire, previously validated for detecting common mental disorders in the Indian setting [6,7], was translated into the local language. | For each variable of interest, give sources of data and details of methods of assessment (measurement). Describe comparability of assessment methods if there is more than one group |
| Bias | Missing data for any single outcome ranged from 0.2-3% for common mental disorder, obesity, and hypertension to 24%-26% for diabetes and high cholesterol. Because were interested in the magnitude of associations across different chronic condition phenotypes, we wanted to include the same participants in each outcome model and thus were forced to exclude 30% of the sample because there missing one or more outcome. We applied inverse probability weighting (IPW) to address bias due to excluding participants with missing data. IPW weights each observation by the inverse of the probability of being analyzed to create a weighted pseudopopulation that resembles the full sample | Describe any efforts to address potential sources of bias |
| Study size | Of the 11,751 participants linkable to the household demographic roster, 10,703 participants had at least one co-residing household member enrolled in the study. Of participants with co-residing household members, 3,181 were excluded because of missing data on one or more outcomes. Thus, a total of 7,522 participants residing in 2,574 households with complete covariates and at least two sampled adults per household were analyzed in the primary analysis. We additionally examined associations of interest among adults with co-residing parents (1,660 dyads in 1,199 households) and spouses (1598 dyads in 1598 households). | Explain how the study size was arrived at |
| Quantitative variables | Each set of logistic regression models estimated the relative odds of having any chronic condition for individuals living with a household member with any chronic condition relative to individuals who were not living with a household member with a chronic condition (i.e., the odds ratio of any chronic condition associated with living with someone who has any chronic condition).  This first set of models was agnostic to the type of relationship between the index participant and co-residing household members aged 18 years and older and included data from all available household members.  A second set of models examined this association among adult children with co-residing parents.  A third set of models examined these associations among spousal dyads. | Explain how quantitative variables were handled in the analyses. If applicable, describe which groupings were chosen and why |
| Statistical methods | (a) We next estimated three sets of logistic regression models that differed by the type of relationship among household members. All models adjusted for included age (in continuous years), sex, education (total years of schooling and college), marital status (married versus not married), religion (Hindu versus not Hindu), and study site, of the index participant whose outcome was being modeled.  (b) We also estimated the associations for having the same chronic condition as another household member for each study site by specifying an interaction term between the exposure variable and the study site. We tested statistical significance of the interaction term using generalized score tests for Type III contrasts.  (c) We applied inverse probability weighting (IPW) to address bias due to excluding participants with missing data. IPW weights each observation by the inverse of the probability of being analyzed to create a weighted pseudopopulation that resembles the full sample. Supplemental Table 1 shows participant characteristics in the total sample and the sample with complete covariates with and without weighting.  (d) All analyses were model-based and accounted for data correlation arising from sampling multiple individuals in the same household and in the same cluster through generalized estimating equations | (*a*) Describe all statistical methods, including those used to control for confounding |
|  |  | (*b*) Describe any methods used to examine subgroups and interactions |
|  |  | (*c*) Explain how missing data were addressed |
|  |  | (*d*) *Cohort study*—If applicable, explain how loss to follow-up was addressed  *Case-control study*—If applicable, explain how matching of cases and controls was addressed  ***Cross-sectional study*—If applicable, describe analytical methods taking account of sampling strategy** |
|  |  | (*e*) Describe any sensitivity analyses |

Continued on next page

| Results | | |
| --- | --- | --- |
| Participants | Of the 11,751 participants linkable to the household demographic roster, 10,703 participants had at least one co-residing household member enrolled in the study. Of participants with co-residing household members, 3,181 were excluded because of missing data on one or more outcomes. Thus, a total of 7,522 participants residing in 2,574 households with complete covariates and at least two sampled adults per household were analyzed in the primary analysis. We additionally examined associations of interest among adults with co-residing parents (1,660 dyads in 1,199 households) and spouses (1598 dyads in 1598 households). | (a) Report numbers of individuals at each stage of study—eg numbers potentially eligible, examined for eligibility, confirmed eligible, included in the study, completing follow-up, and analysed |
|  |  | (b) Give reasons for non-participation at each stage |
|  |  | (c) Consider use of a flow diagram |
| Descriptive data | Table 1 shows the household- and individual-level characteristics of the sample. A total of 2,574 households with 7,522 individuals were analyzed. On average, we observed 2.9 individuals per household (range: 2 to 11 individuals). The mean age of participants was 38 years (range: 18 to 96 years), and 47% were men. The majority of participants were married (78%) and Hindu (94%). While 43% of individuals had at least one chronic condition, this proportion varied from 36% in Mashobra to 50% in Gujarat. The least common chronic condition was high cholesterol (5.1%), and the most common condition was hypertension (23.6%).  Supplemental Table 1 shows missing data by covariate and participant characteristics in the total sample and the sample with complete covariates with and without weighting. | (a) Give characteristics of study participants (eg demographic, clinical, social) and information on exposures and potential confounders |
|  |  | (b) Indicate number of participants with missing data for each variable of interest |
|  |  | (c) *Cohort study*—Summarise follow-up time (eg, average and total amount) |
| Outcome data | See Table 1 | *Cohort study*—Report numbers of outcome events or summary measures over time |
|  |  | *Case-control study—*Report numbers in each exposure category, or summary measures of exposure |
|  |  | ***Cross-sectional study—*Report numbers of outcome events or summary measures** |
| Main results | The main results only show confounder adjusted results; unadjusted results are included in the Supplemental material.  Variable definitions (with category boundaries) are provided in the methods | (*a*) Give unadjusted estimates and, if applicable, confounder-adjusted estimates and their precision (eg, 95% confidence interval). Make clear which confounders were adjusted for and why they were included |
|  |  | (*b*) Report category boundaries when continuous variables were categorized |
|  |  | (*c*) If relevant, consider translating estimates of relative risk into absolute risk for a meaningful time period |
| Other analyses | See Figure 1 | Report other analyses done—eg analyses of subgroups and interactions, and sensitivity analyses |
| Discussion | | |
| Key results | To our knowledge, this is the first study examining the relationship of five prevalent chronic conditions—hypertension, diabetes, obesity, common mental disorder, and high cholesterol—among co-residing adults in India. Irrespective of familial relationship, adults who resided with another adult with any chronic condition had a 30% higher adjusted odds of having one or more chronic condition themselves.  Our findings are largely consistent with prior literature examining the relationship of metabolic outcomes among spouses [16–19] and between parents and adolescent children [17,18,20] within nuclear families. | Summarise key results with reference to study objectives |
| Limitations | Thus, we had relatively small samples in the dyadic analysis, and we interpret tests of statistical significance and differences between the spousal and parent-child dyads with caution. We also lacked sufficient sample size to examine associations in other potential familial relationships of interest (e.g., daughters-in-law and parents-in-law or disaggregating mother-child from father-child). | Discuss limitations of the study, taking into account sources of potential bias or imprecision. Discuss both direction and magnitude of any potential bias |
| Interpretation | At the most superficial level, family history of chronic disease can be used for risk stratification [31]. Our results provide preliminary evidence that targeting households in which one adult has a chronic condition may be an effective way to identify other individuals with chronic conditions and potentially prevent chronic conditions before emerging in India, as has been done among spouses elsewhere [32,33]. | Give a cautious overall interpretation of results considering objectives, limitations, multiplicity of analyses, results from similar studies, and other relevant evidence |
| Generalisability | Site-specific analysis assures us that the findings were consistent across the heterogeneous districts. | Discuss the generalisability (external validity) of the study results |
| Other information | | |
| Funding | The study was funded by the RHN division of Indian Council of Medical Research vide grant RFC NO: RHN/NTF/4/2011-2012 dated 28.03.2012. The funder played no role in the analysis or interpretation of data presented in this study. | Give the source of funding and the role of the funders for the present study and, if applicable, for the original study on which the present article is based |

*Give information separately for cases and controls in case-control studies and, if applicable, for exposed and unexposed groups in cohort and cross-sectional studies.

**Note:** An Explanation and Elaboration article discusses each checklist item and gives methodological background and published examples of transparent reporting. The STROBE checklist is best used in conjunction with this article (freely available on the Web sites of PLoS Medicine at http://www.plosmedicine.org/, Annals of Internal Medicine at http://www.annals.org/, and Epidemiology at http://www.epidem.com/). Information on the STROBE Initiative is available at www.strobe-statement.org.
