# Supplementary material for: Chronic disease concordance within Indian households: A cross-sectional study
Source: PLoS Med. 2017 Sep 29;14(9):e1002395. doi: 10.1371/journal.pmed.1002395 (PMC5621663; doi:10.1371/journal.pmed.1002395)
Supplement: S2 Text — (DOCX) [file pmed.1002395.s007.docx]

**S2 Text. Development of the Statistical Analysis Plan**

Study Context

Data for the present study come from the DISHA trial, a community-based cluster randomized trial designed to test the effectiveness of a community health worker-led lifestyle behavior change on hypertension reduction. The protocol and planned analysis of the DISHA trial have been previously published [1].

The present study is a cross-sectional observational analysis that was conceptualized after the baseline data collection was completed. We were interested in testing the hypothesis that chronic conditions or risk factors in one family member influence the risk for chronic conditions in another family member. We felt that the DISHA trial could provide suitable data because multiple adults were sampled in a single household. Combining the team’s interest in cardiometabolic disease and mental health, we sought to study depression, obesity, diabetes, hypertension, and alcohol use disorders, and a composite of these disorders as outcomes. We identified a set of research questions regarding clustering of these disorder in households.

Analysis Plan prior to commencing the present manuscript

The analyses presented in the current manuscript evolved from the following research question and associated statistical analysis:

1. *What is the fixed effect of family member and neighbor health on individual health?*
   1. *We will randomly select one index individual from each household for this analysis. The Chronic Disease Risk Index will be the primary outcome for this analysis.*
   2. *Each index individual will be assigned a family mean index score (mean minus self) and a neighborhood mean (mean minus self); these mean scores will be treated as “exposures.”*
   3. *We will estimate a set of linear models predicting individual-level risk scores by first adjusting only for age and sex; next adding household sociodemographics and family risk scores; and finally adding community socioeconomic composition and neighborhood risk scores.*

The Chronic Disease Risk Index was conceptualized in the original analysis plan as:

*“We will first develop an index of the 5 factors under study using principal components analysis. The first principal component will be treated as the “Index of Chronic Disease Risk.” Properties of the index will be reported, such as variance explained by the index and factor loadings (correlations between individual factors and the composite measure), and the normality of the distribution. We will also report the mean age and proportion of men within each quintile of the index to aid in interpretation.”*

The “5 factors under study” included: alcohol use measures, depressive symptoms, body mass index, HbA1c, and blood pressure *levels* specified as continuous covariates.

Changes to the Analysis Plan after beginning analysis for the present manuscript

Based on telephonic study team meetings, the following revisions to the analysis plan were made to enhance the rigor and better address the central research question of household concordance of disease:

1. Analyze and report results for individual risk factors as outcomes, rather than using the composite Chronic Disease Risk Index as an outcome.
2. Adopt a binary classification of the biomarkers into chronic condition states, which are of greater interest to our target audience of public health researchers with an interest in intervention.
3. Utilize outcome data from all household members (instead of randomly selecting a pair of adults) by creating an exposure variable describing whether any *other* member of the household has a given chronic condition. This exposure variable can be considered a “mean minus self” analog for the binary setting, and was constructed using a do-loop to assign each individual a household chronic condition exposure status based on the presence of conditions in *other* co-residing household members. To account for including outcome data of multiple individuals in a single household, we utilized a robust variance estimator; this estimator also accounted for multiple households at one site.
4. Stratify the analysis by key relationship types—e.g., husband-wife dyads and parent-child dyads—to provide additional insight on potential mechanisms for the observed results in the overall sample.
5. We removed alcohol use from the outcome list because of its low reported prevalence (<0.5% in women).
6. We added high cholesterol to the outcome list because of its importance in the global disease burden and sufficient numbers in our sample (~6% prevalence).
7. We excluded Shimla data from the depression analysis due to the small number of adults reporting depression (<1%).
8. We also excluded “neighborhood” covariates because this was no longer the focus of our paper. Moreover, the inclusion of site-level fixed effects and a random effect for the cluster accounts for any omitted variable bias due to any potential neighborhood effect.
